# Production of Bixin‐Rich Extracts From Annatto Seeds via Ethanol–Water Extraction Integrated With Ultrafiltration Technology

**DOI:** 10.1002/cbdv.71783

**Published:** 2026-09-30

**Authors:** Karolyna B. Miguel, Vicelma Luiz Cardoso, Miria Hespanhol Miranda Reis

**Affiliations:** ^1^ Chemical Engineering Faculty Federal University of Uberlandia, Santa Mônica Uberlândia Minas Gerais Brazil

**Keywords:** bioactive phytochemicals, extraction/separation, functional properties, health foods, polyphenols

## Abstract

This study investigated the extraction and concentration of bioactive compounds from annatto seeds by integrating ethanol–water extraction with ultrafiltration technology. The co‐extraction of phenolic and carotenoid compounds was achieved by employing a mixture of ethanol and water as solvent. The annatto seed extract presented bixin and total phenolics at concentrations of 1.91 ± 0.02 and 52.31 ± 2.45 mg GAE g^−1^ (dry basis), respectively. Subsequent ultrafiltration through a 5 kDa membrane effectively concentrated the target molecules, with a membrane rejection of 47.53 ± 0.22% for total phenolic and 97.90 ± 0.02% for bixin molecules. Initial permeate flux was 28.59 L h^−1^ m^−2^, stabilizing at a steady‐state flux of 9.26 L h^−1^m^−2^. The observed flux decline was predominantly characterized by a cake formation fouling mechanism at the earlier times of filtration, while the final flux was described by both cake formation and pore blockage mechanisms. This work highlights the potential of membrane technology for producing high‐quality annatto extracts, promoting their broader industrial application.

## Graphical abstract

Ethanol–water extraction followed by 5 kDa ultrafiltration yields a bixin‐rich annatto extract with 97.9% bixin rejection, 94.2% recovery in the retentate, and an enrichment factor of 1.29. Fouling analysis indicates an important contribution from cake formation, highlighting the role of membrane–solute interactions in the selective concentration of bixin.

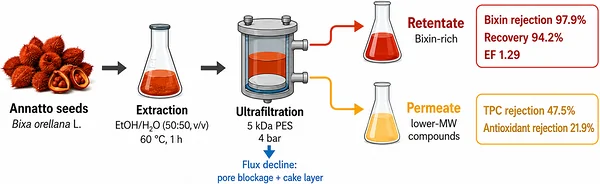

## 1 Introduction

Annatto is a natural and nontoxic reddish pigment derived from the seeds of the achiote tree (*Bixa orellana* (*B. orellana*)), a plant native to Latin America. Annatto seeds are rich in carotenoid pigments, primarily bixin and norbixin. Bixin has been approved by the Food and Drug Administration (FDA) as a food additive and is widely used as an edible colorant in bakery products, ice cream, butter, cheeses, cereals, margarine, and meats [1]. Currently, annatto products represent 11.5% of the global carotenoids market, projected to grow from USD 1.9 billion in 2021 to USD 2.7 billion by 2027 [2]. Beyond its role as a food colorant, bixin exhibits a range of beneficial health properties, including antioxidant, antimicrobial, hypoglycemic, cytotoxic, anti‐inflammatory, and anticancer activities [3, 4]. Consequently, annatto seed extracts can be effectively integrated into food and pharmaceutical products to enhance quality and ensure consumer safety and satisfaction.

The widespread industrial application of bixin depends on the development of effective processes for bixin extraction, concentration and purification [1, 5]. The most commonly used processes for obtaining bixin from annatto seeds involve the use of oil extraction or alkaline aqueous solutions in solid–liquid extraction processes [6, 7, 8, 9]. Some nonconventional extraction processes are reported in the literature to improve the efficiency for bixin extraction, including ultrasound‐assisted [2], microwave‐assisted [10], and supercritical extraction [11] processes. Also, different process conditions are suggested for the extraction of bioactive compounds from annatto seeds [12]. Jayakumar et al. [12] compared the efficiency of ethyl acetate, methanol, and ethanol as solvents for bixin extraction and identified methanol as the optimal solvent by considering the concentration of bixin and total polyphenol compounds and the extract color intensity. However, green solvents are preferred for extracting both lipophilic and hydrophilic fractions from annatto seeds due to their minimized environmental impact, lower process costs, and enhanced food safety. Handayani et al. [13] proposed the use of water as an environmentally friendly and safe solvent for bixin and norbixin extraction from annatto seeds. Shridar et al. [14] presented the optimized conditions of pressure (5 bar), temperature (40°C), process time (10 min), and the seed‐to‐water ratio (1:1) to obtain a maximum yield of 3.41% for bixin extraction. Agudelo Patiño et al. [15] suggested ethanol, a Generally Recognized as Safe (GRAS) substance, as a solvent for bixin extraction from annatto seeds, highlighting its suitability for food‐related applications and its potential as an alternative to more hazardous organic solvents. Strieder et al. [2] also applied ethanol in an ultrasound‐assisted process for bixin extraction.

After the extraction process, a sequential concentration and/or purification step is generally required in a complete process flow of plant‐based products. Membrane separation processes are frequently applied for the purification and concentration of various bioactive compounds due to their low operating costs and high selectivity. Santos et al. [16] demonstrated the efficiency of ultrafiltration in concentrating bioactive compounds from a blackberry pomace aqueous extract by approximately 80%. Seguenka et al. [17] utilized a nanofiltration membrane to concentrate the ethanolic extract of jabuticaba sabará (*Plinia peruviana* (Poir.) Govaerts) peels, reporting retentions greater than 80% for phenolic, flavonoid, and anthocyanin compounds. Vieira et al. [18] evaluated the application of different nanofiltration membranes for anthocyanin concentration from jussara ethanolic extract (*Euterpe edulis*). Doan et al. [19] showed that the ultrafiltration process was efficient for lycopene enrichment in watermelon juice. In our previous work, Miguel et al. [20] applied a polyethersulfone membrane with a molecular weight cut‐off (MWCO) of 10 kDa for the filtration of aqueous annatto seed extract, reporting a 62% retention for phenolic compounds. More recently, Miguel et al. [21] demonstrated that asymmetric alumina hollow‐fiber microfiltration membranes could retain more than 90% of bixin from an aqueous ethanolic annatto seed extract.

Thus, previous studies on annatto seed processing have mainly focused on improving the extraction of bixin and other bioactive compounds through solvent selection or intensified extraction techniques, including ultrasound‐, microwave‐, pressure‐, and supercritical fluid‐assisted processes [2, 10, 11, 12, 13, 14, 15]. However, considerably less attention has been devoted to downstream concentration and fractionation of annatto extracts. Although membrane processes have been successfully applied to concentrate phenolic compounds, anthocyanins, and carotenoids from other plant matrices [16, 17, 18, 19], their application to annatto remains scarcely investigated. In our previous study [20], a 10 kDa polyethersulfone membrane was used to process an aqueous annatto seed extract, with emphasis on the retention of phenolic compounds. However, the low water solubility of bixin limits its recovery in aqueous systems, and the membrane processing of ethanol‐containing annatto extracts, particularly for selective bixin concentration, has not been systematically addressed. Therefore, the novelty of the present work lies in integrating ethanol–water extraction with 5 kDa ultrafiltration to promote the selective concentration of bixin from annatto seed extracts, while simultaneously evaluating the behavior of phenolic and antioxidant compounds. Detailed physicochemical characteristics of the feed and permeate extracts were investigated, with a focus on analyzing the concentrations of key bioactive compounds (bixin concentration, total polyphenolic content, and antioxidant activity). Furthermore, the primary mechanisms of fouling during extract filtration were evaluated by fitting experimental flux data to mathematical models. These inquiries are expected to expand the potential applications of annatto‐derived compounds in various industries.

## 2 Materials and Methods

### 2.1 Material

The annatto (*B. orellana* L.) seeds were obtained as a commercially packaged product (Chamel, KittMed Laboratórios Ltda., Campo Largo, Paraná, Brazil; lot No. 14849). The commercial product label identified the material as seeds of *B. orellana* L. Since the material consisted of commercially packaged seeds and no complete botanical specimen was available for taxonomic authentication, no voucher specimen was deposited. The seeds were stored in dark polyethylene bags at room temperature (approximately 298 K) until further processing and analysis. The extraction was performed with ethyl alcohol (99.8%, Dinâmica, Brazil). Total phenolic content (TPC) analysis utilized Folin–Ciocalteau (Dinâmica, Brazil), sodium carbonate (Dinâmica, Brazil), and gallic acid (Dinâmica, Brazil) reactants. For antioxidant activity assessments, the DPPH assay required 2,2‐diphenyl‐1‐picrylhydrazyl (DPPH) (Sigma–Aldrich, USA), methanol (Vetec, Brazil), and Trolox (Sigma–Aldrich, USA) for calibration. HPLC analysis was performed using acetonitrile (Sigma–Aldrich, Germany), methanol (Dinâmica, Brazil), and a bixin standard (90.0%, Sigma–Aldrich).

### 2.2 Ultrafiltration Process of the Ethanol/Water Annatto Seed Extracts

The ethanol/water annatto seed extract was prepared by conventional extraction using a magnetic stirrer at 150 rpm. The solvent consisted of an ethanol–water mixture (50:50 *v*/*v*). Annatto seeds were added to the solvent at a solid‐to‐liquid ratio of 1:30 (g/mL). Extraction was carried out at 60°C for 1 h, according to conditions suggested in the literature [20]. Following extraction, the seeds were manually removed from the liquid phase, and the extract was filtered through a paper filter (Unifil, Brazil, 12 µm), followed by centrifugation (Beckman, model Coulter Avanti J25) at 5000 rpm for 20 min to remove fine particles.

The ultrafiltration process utilized a commercial flat‐sheet polyethersulfone membrane (Microdyn‐Nadir, UP0005 P) with a MWCO of 5 kDa. According to the manufacturer, the membrane has a total thickness of 210–250 µm, a clean‐water permeance of at least 10 L m^−2^ h^−1^ bar^−1^ under the manufacturer‐specified test conditions, and an operating pH range of 0–14. The selection of membrane cut‐off is an important parameter for achieving the desired concentration of target compounds. The 5 kDa membrane was selected as a compromise between permeate flux and retention of the target compounds. In our previous study [20], a 10 kDa polyethersulfone membrane was applied to an aqueous annatto seed extract; however, the present work focused on an ethanol‐containing extract and specifically aimed to enhance bixin retention. Although the molecular mass of individual bixin molecules is below the nominal MWCO of the membrane, their effective retention may also be influenced by molecular aggregation, solute–membrane interactions, and the development of a fouling layer during filtration. A tighter membrane, such as 1 kDa, would be expected to increase hydraulic resistance and potentially retain a larger fraction of low‐molecular‐weight phenolic compounds, whereas membranes with higher MWCOs, such as 10 or 30 kDa, would favor permeation but could reduce the retention of the target fraction. Therefore, 5 kDa was selected as an intermediate MWCO to balance permeation and selectivity.

The membrane was attached to a stainless‐steel module with a filtration area of 0.0038 m^2^ and pressurized with nitrogen gas. Before all filtration experiments, the membrane was compacted at 4 bar for 30 min using deionized water. Membrane permeability was subsequently determined by measuring the steady‐state flux of pure deionized water and the ethanol–water mixture (50:50 *v*/*v*) across a range of transmembrane pressures (from 1 to 4 bar) at room temperature (approximately 298 K). Dead‐end filtrations of the ethanol/water annatto seed extract were performed at a constant transmembrane pressure of 4 bar and room temperature, achieving a target volume concentration factor of 1.4. The selection of 4 bar as the transmembrane pressure for the extract filtration was based on the preliminary measurements of the membrane permeability. The use of this pressure was also intended to provide an adequate balance between productivity and the expected increase in concentration polarization and fouling at higher pressures. The volume concentration factor (dimensionless) represents the extent of volume reduction during ultrafiltration and was calculated as the ratio between the feed and concentrate volumes.

Figure 1 provides a schematic overview of the experimental workflow, including the extraction and ultrafiltration steps performed in this study.

**FIGURE 1:**
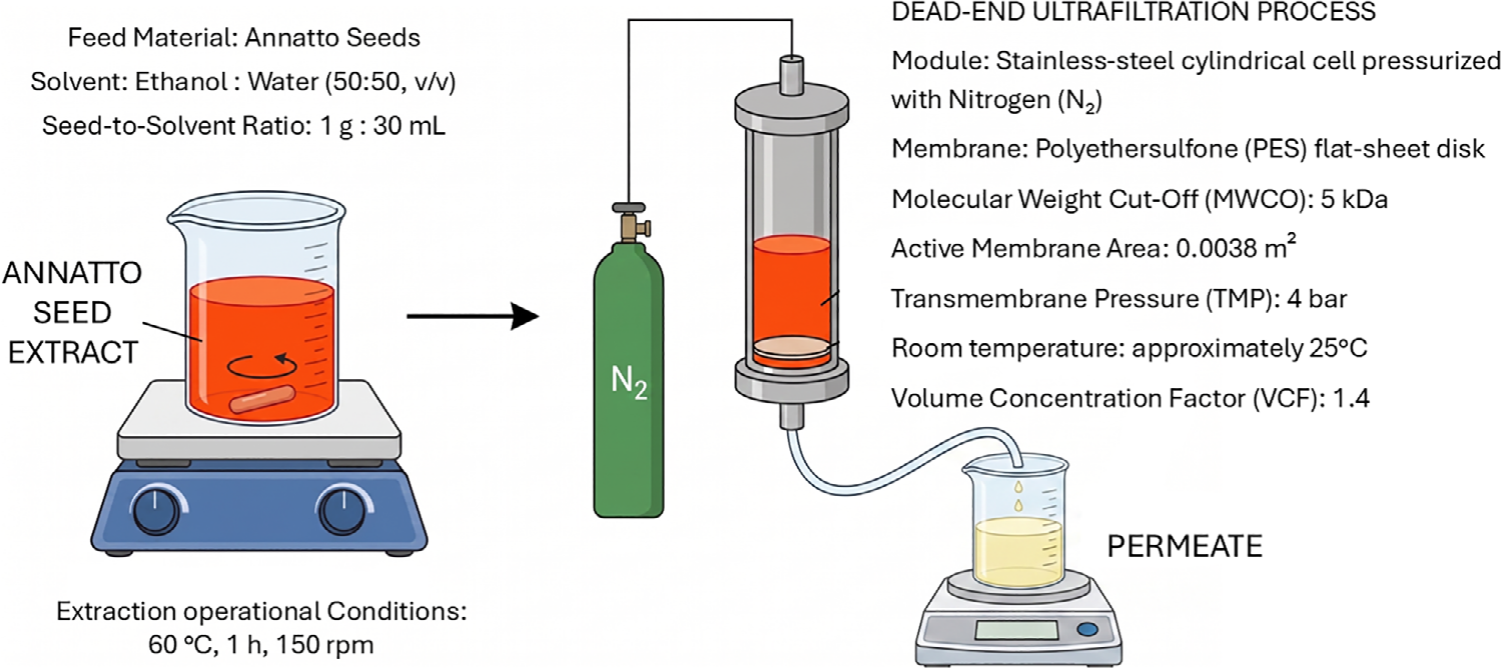
Graphical illustration summarizing the extraction and ultrafiltration steps. Parts of the illustrative elements were generated with the assistance of OpenAI ChatGPT. The final figure was reviewed and edited by the authors.

Experimental flux data were adjusted to the flux decay model proposed by Hermia [22] (Equation 1), as suggested in the literature for dead‐end membrane filtrations [23]. The predominant fouling mechanism was determined by assessing the coefficient of determination (*R* ^2^) derived from comparing numerical simulations with experimental data. The parameter “*n*” was set according to the predominant fouling mechanism: *n* = 0 for cake formation; *n* = 1 for intermediate pore blockage, *n* = 1.5 for standard pore blockage and *n* = 2 for complete pore blockage. Also, the optimal parameter “*n*” was identified through fitting experimental data to Equation (1).

(1) $$-\;\left(\frac{dJ}{dt}\right)=K_{n}\;J^{3-n}\;$$

where *J* is the permeate flux (L h^−1^ m^2^), t is the filtration time (h), and *K*_n_ is an adjusted parameter.

Fouling analyses during the ultrafiltration of annatto seed extract were also considered according to the series resistance model. The total hydraulic resistance (*R*_t_) was considered as the sum of the intrinsic membrane resistance (*R*_m_), pore‐blocking resistance (*R*_p_), and cake‐layer resistance (*R*_c_). The intrinsic membrane resistance was calculated from the pure‐water flux through the clean membrane, as presented in Equation (2).

(2) $$R_{m}=\frac{\Delta P}{\mu J_{w,0}}\;$$

where $J_{w,0}$ is the pure‐water permeate flux through the clean membrane (m^3^ m^−2^ s^−1^), ΔP is the transmembrane pressure (Pa), and μ is pure‐water the dynamic viscosity (Pa·s).

After filtration of the annatto extract, the total resistance was calculated from the pure‐water flux through the fouled membrane before removal of the cake layer (*J*_w,f_), as presented in Equation (3).

(3) $$R_{t}=\frac{\Delta P}{\mu J_{w,f}}\;$$

where $J_{w,f}$ is the pure‐water permeate flux through the fouled membrane (m^3^ m^−2^ s^−1^).

After gently removing the surface cake layer, the remaining resistance was determined from the pure‐water flux through the partially cleaned membrane (*J*_w,c_), as presented in Equation (4).

(4) $$R_{p}+R_{m}=\frac{\Delta P}{\mu J_{w,c}}\;$$

where $J_{w,c}$ is the pure‐water permeate flux through the partially cleaned (after cake removal) membrane (m^3^ m^−2^ s^−1^).

The pore‐blocking resistance was obtained by subtracting the intrinsic membrane resistance (Equation 2) from the resistance measured after removal of the cake layer (Equation 4). The cake‐layer resistance was subsequently calculated by subtracting the membrane and pore‐blocking resistances from the total resistance (Equation 3).

### 2.3 Extract Characterizations

Annatto seed extracts, both before and after the ultrafiltration process, were characterized for bixin concentration, total phenolic compounds (TPC) content, antioxidant activity (determined by the DPPH method), and color. Color analyses of the samples were performed using a Konica Minolta CR‐5 digital colorimeter.

Bixin content in the extract samples was quantified by high‐performance liquid chromatography (HPLC) using a Shimadzu LC‐20AT Prominence system equipped with a CBM‐20A controller, an automatic liquid sampler (ALS), an SPD‐20AV UV–vis detector, and a VP‐ODS C18 column (150 mm × 4.6 mm, 3 µm). Following the methodology proposed by Monge Neto et al. [24], dried extract samples were diluted in acetonitrile, and a 20 µL sample volume was injected. The chromatographic separation was performed at a flow rate of 1.0 mL min^−1^ for a running time of 30 min. The mobile phase consisted of a gradient program using acetonitrile (eluent A) and 2% (*v*/*v*) acetic acid in water (eluent B), starting with an isocratic elution of 65% A and 35% B. The column temperature was maintained at 29°C. Bixin was monitored at 470 nm. Quantification was achieved using a bixin calibration curve, and results were expressed as milligrams of bixin per gram of dried extract (mg bixin g^−1^ dry extract). Bixin standard solutions, ranging in concentrations from 2 to 14 mg L^−1^, were used to construct a calibration curve, yielding LOD and LOQ values of 0.04 and 0.12 mg L^−1^, respectively. The calibration equation was *y* = 32095 x, where *y* represents the chromatographic peak area and x represents the bixin concentration (mg L^−1^), with a coefficient of determination of *R* ^2^ = 0.986. Under these chromatographic conditions, bixin showed a retention time of approximately 13 min. The chromatograms for the standard solutions are presented in the Supporting Information (Figure 1S).

Total phenolic compounds were determined spectrophotometrically using the Folin–Ciocalteu colorimetric method, according to previously reported procedures [20, 25, 26, 27]. Briefly, a 150 µL aliquot of each extract was mixed with 150 µL of Folin–Ciocalteu reagent and 2700 µL of sodium carbonate, resulting in a final reaction volume of 3.0 mL. The reaction mixture was homogenized and incubated for 30 min before spectrophotometric analysis. Absorbance was then measured at 725 nm using a Shimadzu UV‐1280 spectrophotometer. A calibration curve was prepared using gallic acid as the reference phenolic compound, and the concentration of TPC was calculated from the corresponding calibration curve and expressed as milligrams of gallic acid equivalent (GAE) per gram of dried extract (mg_GAE_ g^−1^ dry extract). Feed, permeate, and concentrate samples were analyzed in triplicate for each independent extraction–ultrafiltration experiment.

The antioxidant activity values of the annatto seed extracts were determined using the DPPH (2,2‐diphenyl‐1‐picrylhydrazyl) radical scavenging assay by measuring the solution absorbance at 595 nm after mixing the extract with the DPPH reagent [20]. After the reaction period, the decrease in absorbance resulting from the reduction of the DPPH radical by antioxidant compounds present in the extracts was determined spectrophotometrically using a Shimadzu UV‐1280 spectrophotometer. A calibration curve was prepared using a Trolox solution as a standard, and the antioxidant activity of the feed, permeate, and concentrate samples was calculated from the corresponding calibration curve. Results were expressed as micromoles of Trolox equivalent (TE) per gram of dried extract (µmol_TE_ g^−1^ dry extract). The analyses were performed in triplicate for each independent extraction–ultrafiltration experiment.

Membrane rejection (MR, %) was used to evaluate the efficiency of the membrane in retaining the compounds of interest and was calculated according to Equation (5).

(5) $$\mathrm{MR}\;=\left(1\;-\frac{C_{p}}{C_{f}}\right)\;\times \;100\;$$

where *C*_f_ and *C*_p_ are the concentrations of the target compound in the feed and permeate, respectively.

In addition to membrane rejection, a mass balance was performed considering the concentrations of each compound in the feed, permeate, and concentrate streams and the corresponding stream volumes. The recovery (*R*, %) of each compound in the concentrate was calculated from the total amount of the compound initially present in the feed and the amount recovered in the final concentrate, as described by Equation (6).

(6) $$R=\left(\frac{m_{c}}{m_{f}}\right)\;\times \;100\;$$

where m_c_ and m_f_ are the masses of the target compound in the concentrate and feed, respectively.

Finally, the enrichment factor (EF) was used to quantify the increase in the concentration of each compound in the concentrate relative to the initial feed and was calculated according to Equation (7):

(7) $$\mathrm{EF}\;=\left(\frac{C_{c}}{C_{f}}\right)\;$$

where *C*_c_ is the concentration of the target compound in the concentrate.

Two independent extraction–ultrafiltration experiments (*n* = 2) were performed, with a fresh annatto seed extract independently prepared for each filtration experiment. Experimental flux values are expressed as mean ± standard deviation of two independent filtration experiments (*n* = 2). For each independent experiment, the physicochemical analyses of the feed, permeate, and concentrate samples were performed in triplicate as technical replicates (*n* = 3). The technical replicates were averaged within each independent experiment, and the results are expressed as the mean ± standard deviation of the two independent experimental replicates. Technical replicates were not treated as independent observations for statistical inference.

## 3 Results and Discussion

### 3.1 Flux Analyses of Membrane Ultrafiltration Processes

Figure 2 illustrates the linear relationship between experimental flux data and transmembrane pressure for both pure water and the ethanol–water solution (50:50 *v*/*v*) filtered through the ultrafiltration membrane, with *R* ^2^ values greater than 0.99. The linear flux‐pressure relationship is consistent with Darcy's law. The water permeance through the ultrafiltration membrane was calculated to be 27.31 ± 0.90 L h^−1^ m^−2^ bar^−1^, exceeding the minimum clean‐water permeance of 10 L h^−1^ m^−2^ bar^−1^ specified by the manufacturer. This permeance slightly decreased to 25.80 ± 0.59 L h^−1^ m^−2^ bar^−1^ when filtering the ethanol–water solution. Solvent properties like polarity and viscosity influence membrane permeability, and the higher polarity of water and the hydrophilic nature of the polyethersulfone membrane likely contributed to the improved water flux compared to that of the ethanol–water mixture.

**FIGURE 2:**
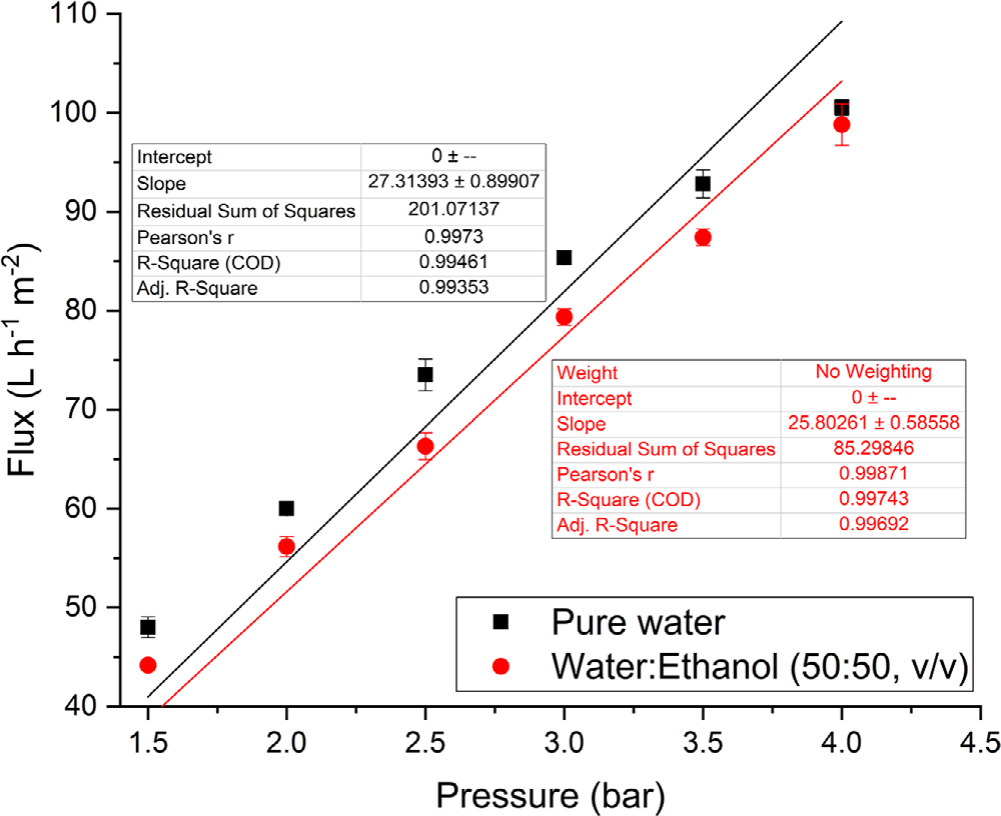
Experimental permeate flux of pure water and ethanol–water mixture (50:50, *v*/*v*) through the 5 kDa membrane as a function of transmembrane pressure.

Figure 3a illustrates the experimental and calculated flux decline over time during the ultrafiltration of the annatto seed extract using the 5 kDa membrane. The process was conducted at a transmembrane pressure of 4 bar for 140 min. We observed a decrease in permeate flux within the initial 10 min of filtration, after which the flux stabilized, reaching a steady‐state permeate flux of 9.26 ± 0.62 L h^−1^ m^−2^. This decline in permeate flux can be attributed to concentration polarization and fouling phenomena. For comparison, Conidi et al. [28] reported a lower final permeate flux of approximately 2 L h^−1^ m^−2^ when purifying and concentrating bioactive compounds from goji plant (*Lycium barbarum* L.) using a 4 kDa polyethersulfone membrane. Similarly, Sánchez‐Arévalo et al. [29] observed a final flux of 4.1 L h^−1^ m^−2^ during the recovery of bioactive compounds from olive pomace extracts with a 5 kDa polymeric membrane. In our previous work, Miguel et al. [20] reported a higher final permeate flux of 24.02 ± 0.16 L h^−1^ m^−2^ for the filtration of aqueous annatto seed extract using a flat polyethersulfone membrane with a MWCO of 10 kDa. This was achieved under cross‐flow filtration mode, with a transmembrane pressure of 6 bar, a cross‐flow velocity of 8.20 cm s^−1^, and a concentration factor of 3.0. Although Miguel et al. [20] applied a relatively small cross‐flow velocity, the cross‐flow filtration mode itself contributes to fouling mitigation and an increase in permeate flux compared to the dead‐end mode used in the current study. Furthermore, the higher MWCO of the membrane used by Miguel et al. [20] would naturally lead to a higher permeate flux. Additionally, our current study employed ethanol as a solvent to enhance the concentration of bioactive compounds in the extract, whereas Miguel et al. [20] filtered an aqueous annatto seed extract. The presence of ethanol, along with the hydrophilic characteristic of the polyethersulfone membrane, likely contributed to the increased membrane fouling observed in this study, consequently leading to a decrease in permeate flux compared to the aqueous system.

**FIGURE 3:**
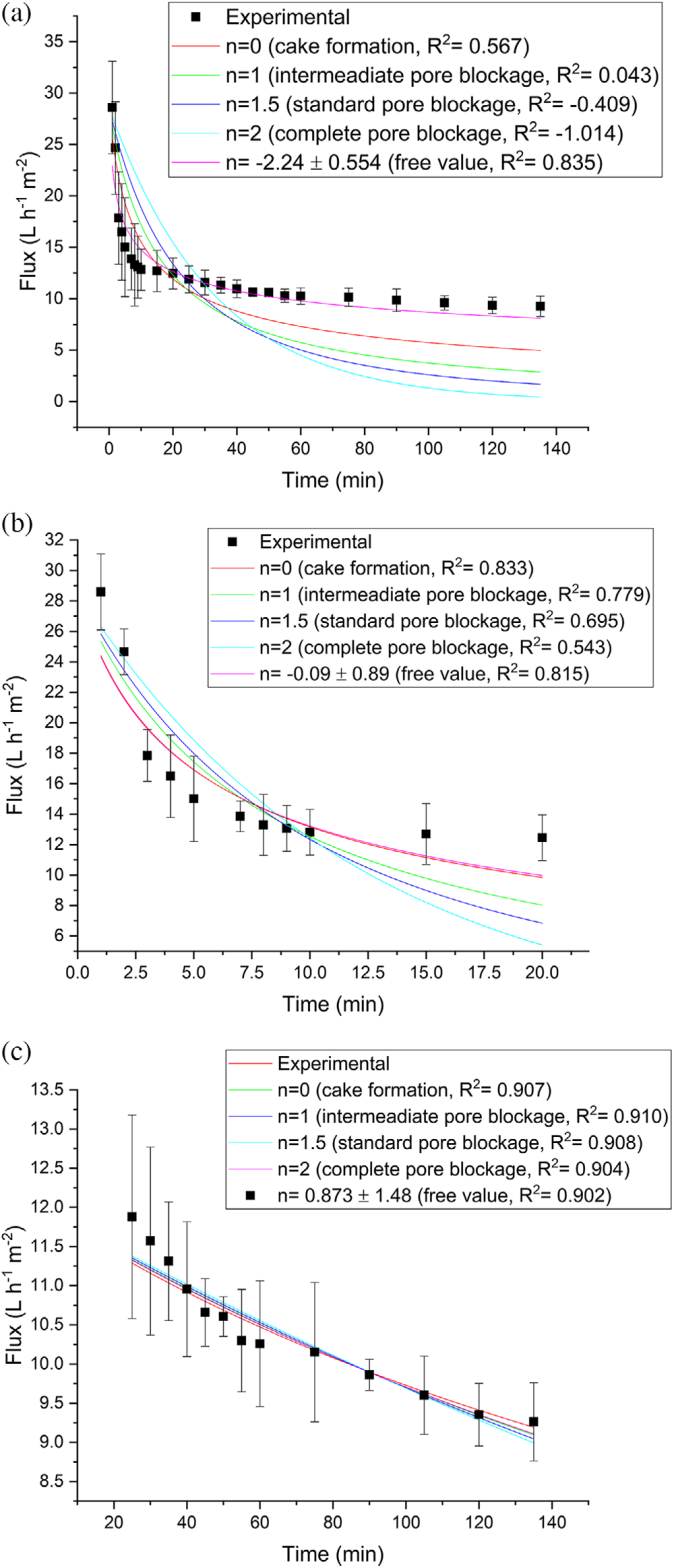
Permeate flux decline and fouling analysis during the ultrafiltration of annatto seed extract using a 5 kDa membrane at 4 bar. (a) Full filtration profile (0–140 min); (b) initial filtration stage (0–20 min); and (c) final filtration stage (20–140 min).

The experimental permeate flux showed a pronounced decline during the ultrafiltration of the annatto seed extract, particularly within the first 10 min of filtration, as shown in Figure 3a. Initially, the experimental data over the entire 140‐min filtration period were fitted to the Hermia model using each of the standard *n* values associated with the classical fouling mechanisms (*n* = 0 for cake formation; *n* = 1 for intermediate pore blockage, *n* = 1.5 for standard pore blockage and *n* = 2 for complete pore blockage). However, none of these individual mechanisms provided an adequate description of the experimental flux decline over the entire filtration period. Therefore, *n* was subsequently treated as a free fitting parameter to determine whether an intermediate value could better represent the overall filtration behavior. This adjustment resulted in a negative *n* value. Since negative *n* values have no conventional physical interpretation within the Hermia model, this result should not be considered evidence of a distinct fouling mechanism. Instead, it indicates that the complete filtration curve cannot be adequately represented by a single fouling mechanism or by a single constant *n* value. The markedly different flux behavior observed between the initial rapid decline and the subsequent stabilization period suggests that the predominant fouling mechanisms evolved throughout filtration. Therefore, the filtration data were subsequently divided into different time intervals to identify the mechanisms governing each stage of the process.

Then, to better understand the predominant fouling mechanisms, the filtration data was divided into two distinct sections: the initial 20 min, representing the most pronounced flux decline (Figure 3b), and the subsequent period from 20 to 140 min, which corresponds to the flux stabilization phase (Figure 3c). For the initial 20 min of filtration (Figure 3b), cake formation (*n* = 0) was identified as the predominant fouling mechanism, providing the best fit for the Hermia model with an *R* ^2^ of 0.833. The intermediate pore blockage model (*n* = 1) also showed a relatively good fit (*R* ^2^ = 0.779), whereas the other pore‐blocking models presented *R* ^2^ values below 0.70. The free adjustment for this period also yielded an *n* value of −0.09 ± 0.89, which is close to the theoretical value of *n* = 0 associated with cake filtration. However, the relatively large uncertainty for the free *n* value indicates that intermediate pore blockage (*n* = 1) may also have contributed to the initial filtration stage. Therefore, cake formation appears to have made a greater contribution during the initial filtration stage, while intermediate pore blockage may also have occurred. Cake formation is commonly observed in dead‐end filtration, particularly when suspended particles or aggregates are larger than the membrane pores. These larger particles and aggregates may also partially obstruct the pore entrances, contributing to intermediate pore blockage during the initial filtration period.

According to the literature [30], annatto seeds contain significant amounts of lipids, proteins, and total carbohydrates, and these macromolecules probably contribute to the formation of a cake layer on the membrane surface. For the later stage of filtration (from 20 to 140 min, Figure 3c), the evaluated Hermia models provided similar fits to the experimental data, with *R* ^2^ values close to 0.9. The free adjustment resulted in an *n* value of 0.873 ± 1.48. The large uncertainty associated with the fitted *n* value encompasses the full range of *n* values from 0 to 2, preventing the unambiguous identification of a single predominant mechanism during this filtration stage. Therefore, the results suggest that both cake formation and pore‐blocking mechanisms may have contributed to the overall membrane fouling. The applied transmembrane pressure of 4 bar likely contributed to the ingress of some molecules into the membrane pores after the cake formation, thereby making pore blockage mechanisms more pronounced.

Fouling during the ultrafiltration of annatto seed extract was also analyzed using the series resistance model. Analysis of hydraulic membrane resistance (*R*_m_), cake resistance (*R*_c_), and pore blockage resistance (*R*_p_) yielded values of (0.98 ± 0.002), (1.07 ± 0.001), and (0.86 ± 0.003) 10^12^ m^−1^, respectively. Considering the total fouling resistance (*R*_c_ + *R*_p_), the cake layer accounted for approximately 55.4%, while pore blockage contributed 44.6%, indicating a slightly greater contribution of cake formation to the overall fouling resistance. The calculated membrane resistance is in close agreement with the values reported by Miguel et al. [20] for filtering an aqueous annatto seed extract through a 10 kDa polyethersulfone membrane.

### 3.2 Physicochemical Characteristics of Feed, Permeate, and Concentrate Extracts

The physicochemical characteristics of the annatto seed extract before and after the proposed filtration process are shown in Table 1. Values are expressed as mean ± standard deviation of two independent extraction–ultrafiltration experiments (*n* = 2). For each independent experiment, analytical measurements were performed in triplicate (technical replicates, *n* = 3). Technical replicates were averaged before calculating the mean and standard deviation between independent experiments. The annatto seed extract exhibited 2.04 ± 0.49 mg_GAE_ per gram of seeds for TPC, which is equivalent to 52.31 ± 2.45 mg_GAE_ g^−1^ (dry basis). Total phenolic content in annatto seed extracts exhibits considerable variability across different studies, primarily influenced by the extraction methodology and source material characteristics. Jayakumar et al. [12] observed a range of TPC values (0.018 to 5.1 mg_GAE_ per gram of seeds) with different extraction conditions (solvent, temperature, solid: liquid ratio, and time), noting a maximum of 5.1 mg_GAE_ g^−1^ when using methanol at 70°C with a 1:15 seed‐to‐solvent ratio over 20 min. Miguel et al. [20] obtained a TPC of 2.02 ± 0.01 mg_GAE_ per gram of seeds from an aqueous annatto extract. Quintero Quiroz et al. [31] applied a response surface design to optimize the extraction conditions under ultrasound irradiation with annatto seeds and reported the maximum TPC value of 3.81 mg_GAE_ per gram of seeds at pH of 7.0, a seed‐to‐solvent ratio of 1:7, and a treatment time of 20 min with ethanol as solvent. Agudelo Patiño et al. [15] presented a TPC value of 5.45 ± 3.12 mg_GAE_ per gram of seeds for the annatto seed extract. The permeability of the membrane to phenolic compounds is likely influenced by both its porosity and the relatively small size of these compounds, which typically range from 200 to 3500 Da. Due to their low molecular weight, phenolic compounds can more easily pass through the membrane compared to larger molecules, contributing to their permeation rate.

**TABLE 1:** Physico‐chemical characteristics of the annatto seed extracts before and after ultrafiltration.

Parameter	Annatto extracts		
	Feed	Final concentrate	Final permeate
Total phenolic content (mg_GAE_ g^−1^, dry basis)	52.31 ± 2.45	57.60 ± 2.70	27.44 ± 1.17
Antioxidant activity (µmol_TE_ g^−1^, dry basis)	365.9 ± 9.7	199.7 ± 6.1	285.6 ± 2.1
Bixin concentration (mg g^−1^, dry basis)	1.91 ± 0.02	2.47 ± 0.12	0.04 ± 0.00
Color coordinate L*	79.22 ± 0.15	83.27 ± 1.76	92.47 ± 1.22
Color coordinate a*	8.67 ± 0.13	10.34 ± 0.08.	−2.85 ± 0.04
Color coordinate b*	71.72 ± 0.02	22.68 ± 0.45	26.86 ± 1.33

The ethanol/water extract exhibited substantial antioxidant activity. The antioxidant activity of the produced ethanol/water extract is similar to that reported by Quintero Quiroz et al. [31]. The membrane rejection of antioxidant compounds was 21.93 ± 0.40%., which aligns with the retention observed for the phenolic molecules.

Bixin is the primary carotenoid pigment in annatto, and its concentration varies widely based on extraction methods and seed characteristics. According to chromatographic analyses, the annatto seed extract presented almost 2 mg of bixin per gram of dried sample. Sudha et al. [10] reported a bixin concentration of approximately 6 mg g^−1^ by using a microwave‐assisted process. Representative chromatograms of the feed, permeate, and concentrate samples are presented in Figures S2 and S3. In addition to the peak assigned to bixin, observed at a retention time of approximately 13 min (Figure S3), several other chromatographic peaks were detected, indicating the presence of additional compounds in the ethanol/water annatto seed extract. Previous studies using HPLC‐DAD‐MS/MS and LC/ESI‐MS have demonstrated that annatto seed extracts may contain several phenolic compounds and flavonoids in addition to bixin. Chisté et al. [32] identified hypolaetin and a caffeoyl acid derivative as major phenolic compounds in annatto seeds, whereas Quintero Quiroz et al. [31] reported compounds such as catechin, chlorogenic acid (5‐caffeoylquinic acid), chrysin, butein, hypolaetin, licochalcone A, and xanthohumol in annatto seed extracts. Therefore, some of the additional peaks observed in Figure S2 may be associated with phenolic compounds, flavonoids, carotenoid‐related compounds, or other extract constituents. Future studies using LC–MS/MS or high‐resolution mass spectrometry should be performed to identify these compounds specifically in the ethanol–water annatto seed extract and to evaluate their behavior during ultrafiltration.

The applied extraction conditions may influence the measured bixin concentration, since bixin is susceptible to isomerization and degradation depending on temperature, exposure time, and solvent composition. Bixin is a naturally occurring cis‐carotenoid that may undergo cis–trans isomerization and thermal or oxidative degradation depending on the processing conditions. Rios et al. [33] demonstrated that heating bixin in a water–ethanol medium promoted its degradation and the formation of di‐cis and all‐trans isomers, as well as a C17 degradation product. In the present study, extraction was performed using an ethanol–water mixture (50:50 *v*/*v*) at 60°C for 1 h. Therefore, some degree of bixin isomerization or degradation during extraction cannot be completely excluded. However, the temperatures investigated by Rios et al. [33] ranged from 70°C to 125°C, which are higher than the extraction temperature used in the present work. Moreover, the subsequent ultrafiltration step was conducted at room temperature (approximately 298 K), without additional heating, thereby minimizing the likelihood of further thermally induced transformations during membrane processing.

The ultrafiltration process was particularly efficient for bixin concentration. For bixin, the membrane rejection was 97.90 ± 0.02%, while approximately 94.2% of the initial bixin mass was recovered in the concentrate. The bixin EF was 1.29 at a volume concentration factor of 1.4. This value is consistent with the high carotenoid retentions reported for other membrane‐based processes involving carotenoid‐rich plant matrices. Miguel et al. [21] reported bixin retention higher than 90% during the microfiltration of an ethanol–water annatto seed extract using asymmetric ceramic hollow‐fiber membranes. Similarly, Doan et al. [19] observed lycopene rejection above 94% during the ultrafiltration of watermelon juice with a 1 kDa membrane, whereas Paes et al. [34] reported lycopene retentions higher than 90% during ultrafiltration of papaya pulp using 50 and 100 kDa membranes. High carotenoid rejection has also been observed for other plant extracts. Conidi et al. [28] reported β‐carotene rejection above 90% during ultrafiltration of Goji berry extract, while Machado et al. [35] obtained approximately 98% carotenoid rejection from pequi aqueous extract by ultrafiltration. Figure 4 presents the membrane rejection for the evaluated compounds from the annatto seed extract, clearly illustrating the high selectivity for bixin compared to the moderate permeation of phenolic and antioxidant compounds.

**FIGURE 4:**
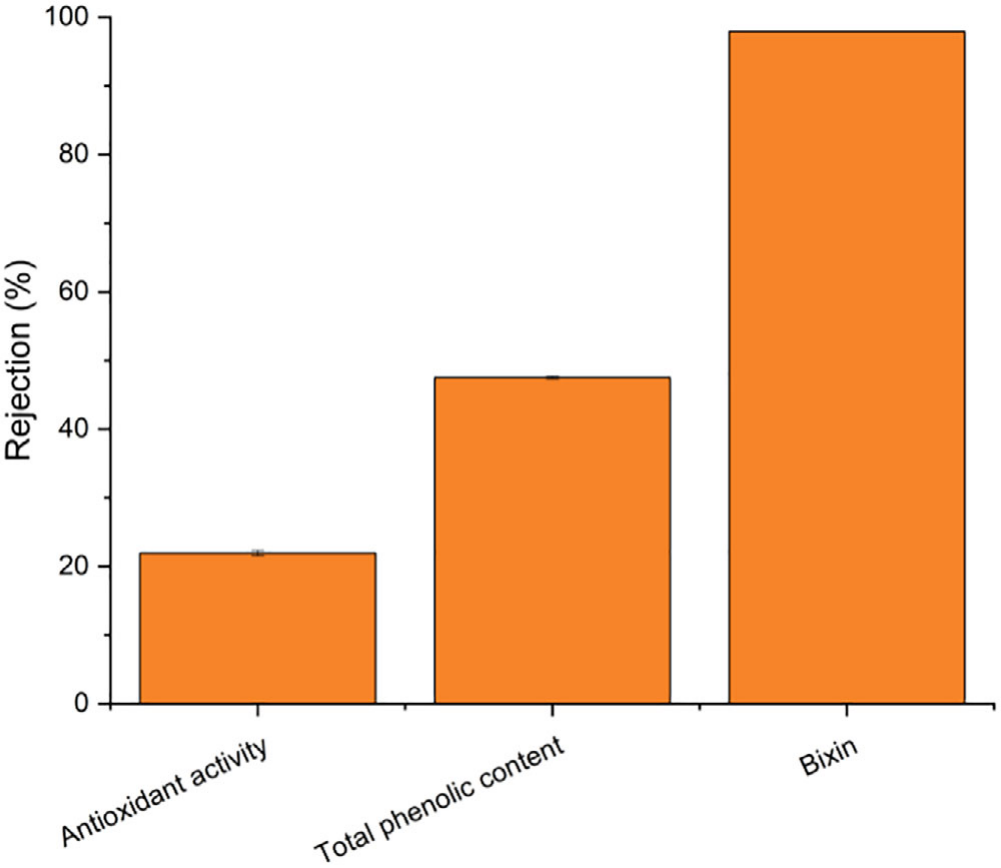
Membrane rejection for total phenolic, antioxidant, and bixin compounds from annatto seed extract.

The exceptionally high bixin retention cannot be attributed solely to molecular size exclusion. The molecular weight of bixin (∼394.5 Da) is considerably lower than the nominal MWCO of the membrane (5 kDa), indicating that additional separation mechanisms must contribute to its retention. One possible mechanism is molecular association or aggregation. Bixin has a long‐conjugated hydrocarbon chain and a predominantly hydrophobic character, and previous studies have demonstrated that bixin can form molecular aggregates in polar solvent–water mixtures [36]. Therefore, although individual bixin molecules are sufficiently small to permeate through the membrane pores, aggregation may increase their effective hydrodynamic size and consequently enhance their retention. In addition, bixin may associate with larger components present in the annatto extract. Annatto seeds contain lipids, proteins, carbohydrates, and other macromolecular or colloidal constituents [30], which may form complexes or aggregates with carotenoid molecules. Bixin may also interact with the membrane surface and, particularly, with the organic material progressively deposited during filtration. Such membrane–solute and solute–deposit interactions can further decrease the effective transport of bixin through the membrane.

The potential contribution of the progressively formed fouling layer to the high apparent membrane retention was further investigated through a time‐resolved evaluation of permeate composition. Figure 5 presents the TPC (Figure 5a) and antioxidant activity (Figure 5b) of the permeate samples collected throughout the filtration process. Both parameters exhibited a distinct upward trend as filtration time progressed. This behavior is likely associated with the gradual passage of fine particles and soluble phenolic compounds through the membrane as filtration time increases. As the fouling layer forms and compacts on the membrane surface, smaller particles and dissolved compounds that were initially retained can eventually permeate, contributing to the higher phenolic content and antioxidant activity observed. These quantitative findings are corroborated by the visual assessment presented in Figure 5c, where a progressive intensification of the yellow coloration is evident. This chromatic change confirms the increased permeation of pigment‐rich compounds (likely associated with carotenoids from the annatto seed) and aligns consistently with the measured increase in bioactive content.

**FIGURE 5:**
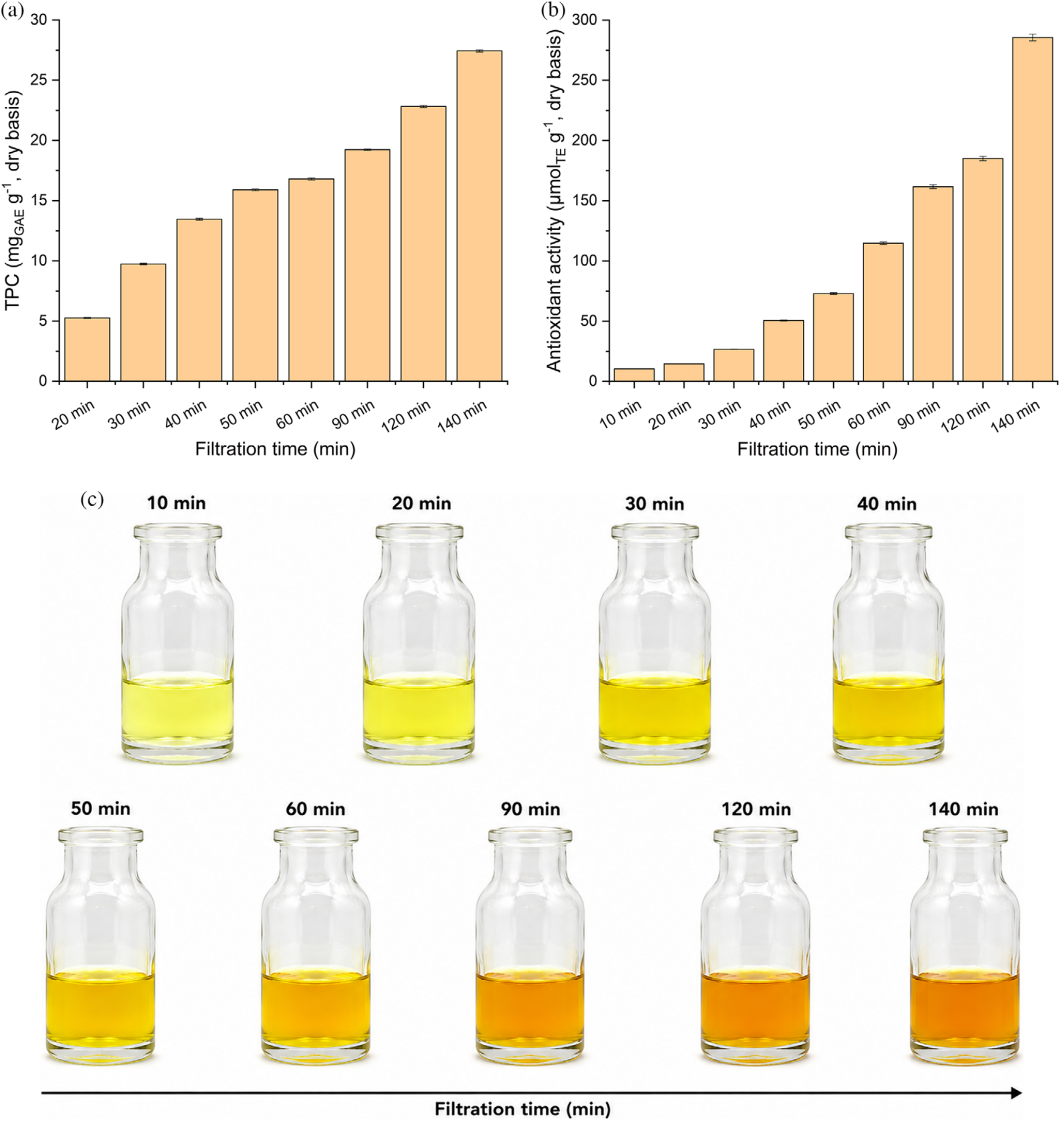
Characterization of permeate samples as a function of filtration time: (a) total phenolic content; (b) antioxidant activity; and (c) visual appearance of the collected fractions. For panel (c), OpenAI ChatGPT was used solely to remove the image background and assist with the arrangement of the original photographs. No modifications were made to the appearance, color, shape, or content of the experimental samples.

The apparent increase in the concentration of TPC and antioxidant activity in the permeate over time can be explained by considering the dynamic nature of the dead‐end ultrafiltration process and the physicochemical interactions occurring at the membrane interface. As filtration progresses, two phenomena become increasingly relevant. First, the concentration of solutes in the feed solution rises due to continuous solvent removal, increasing the driving force for mass transfer across the membrane. This effect is particularly pronounced for low‐molecular‐weight phenolic compounds and antioxidant molecules. Due to their relatively small size and hydrophilic nature, these compounds typically range from 200 to 3500 Da and exhibit partial permeability through the 5‐kDa polyethersulfone membrane [18, 37]. Second, the evolution of the fouling layer on the membrane surface plays a critical role. Initially, the formation of a cake layer of macromolecular aggregates and suspended solids enhances the membrane selectivity by providing an additional barrier to solute transport. However, as this layer compacts under sustained transmembrane pressure, its structure may become denser yet more heterogeneous, potentially allowing the passage of smaller solutes that were previously retained [23]. The structural rearrangement of the fouling layer coupled with increasing solute concentrations in the feed results in a gradual increase in permeate concentrations of TPC and antioxidants. This behavior reflects the temporal evolution of membrane performance and solute‐membrane interactions under non‐steady‐state conditions.

Figure 6 shows the visual appearance of the feed, permeate, and concentrate samples at the final filtration, together with the corresponding amounts of bioactive compounds calculated based on the volume of each stream. The mass balance was performed to quantify the distribution of bioactive compounds after ultrafiltration, using the applied concentration factor of 1.4 and the measured concentrations on a volumetric basis. The concentration factor defined the relative stream volumes: the feed volume was set to 1.4 units, the concentrate to 1.0 unit, and the permeate to 0.4 units. According to mass balance principles, the total mass of a compound entering the system in the feed must equal the mass leaving in the concentrate and permeate streams plus any fraction retained on or adsorbed to the membrane. For each stream, the mass of a given compound was obtained by multiplying its volumetric concentration by the corresponding relative volume.

**FIGURE 6:**
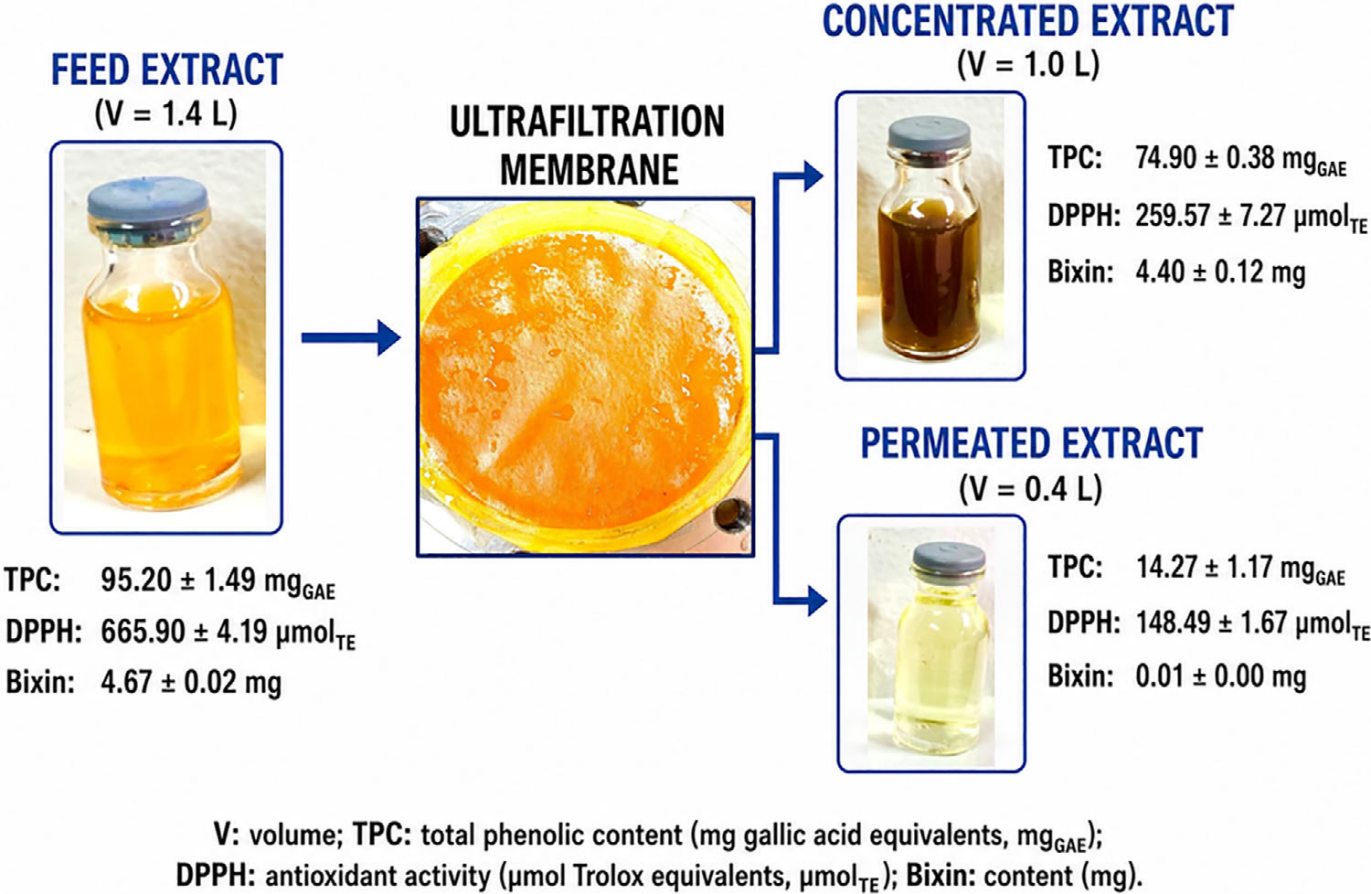
Visual appearance and mass balance of bioactive compounds in the feed, permeate, and concentrate streams during the ultrafiltration process. OpenAI ChatGPT was used solely to remove the image backgrounds and assist with the arrangement of the original photographs. No modifications were made to the appearance, color, shape, or content of the experimental samples or membrane.

According to the data presented in Figure 6, the residual mass, which likely corresponds to the fraction adsorbed onto the membrane, was calculated as 6.41 ± 0.09% for TPC and the unaccounted fraction based on antioxidant activity was 38.7 ± 0.3%. For bixin, the overall mass‐balance closure was approximately 94.6%, indicating that 5.40 ± 0.01% of the initial bixin mass was not accounted for in the concentrate and permeate streams. This unaccounted fraction may be associated with adsorption or deposition of bixin on the membrane and cake layer, as well as with experimental and analytical uncertainties. The color of the extract is directly related to its carotenoid content. The ultrafiltration process yielded a clear, transparent permeate, while the feed extract presented yellowish color coordinates. The permeate displayed higher luminosity (L*) and a notable reduction in the a* coordinate (associated with redness), along with a reduced yellowish color (associated with the b* coordinate). A more intense reddish color was observed in the concentrate stream due to the increase in the a* coordinate.

After filtration, a yellow cake layer was visually observed on the membrane surface, confirming the retention of carotenoid compounds. SEM analysis provided direct morphological evidence of membrane fouling after ultrafiltration (Figure 7). Comparison of the cross‐sectional images of the clean and used membranes revealed the formation of a dense cake layer on the membrane surface, with an average thickness of 24.25 ± 1.94 µm. In addition, the outer‐surface image of the used membrane showed extensive deposition of approximately spherical particles and aggregates, with characteristic dimensions of approximately 3 µm. These observations confirm the substantial accumulation of material at the membrane surface during filtration and are consistent with the fouling analysis, which indicated an important contribution of cake formation to the overall filtration resistance.

**FIGURE 7:**
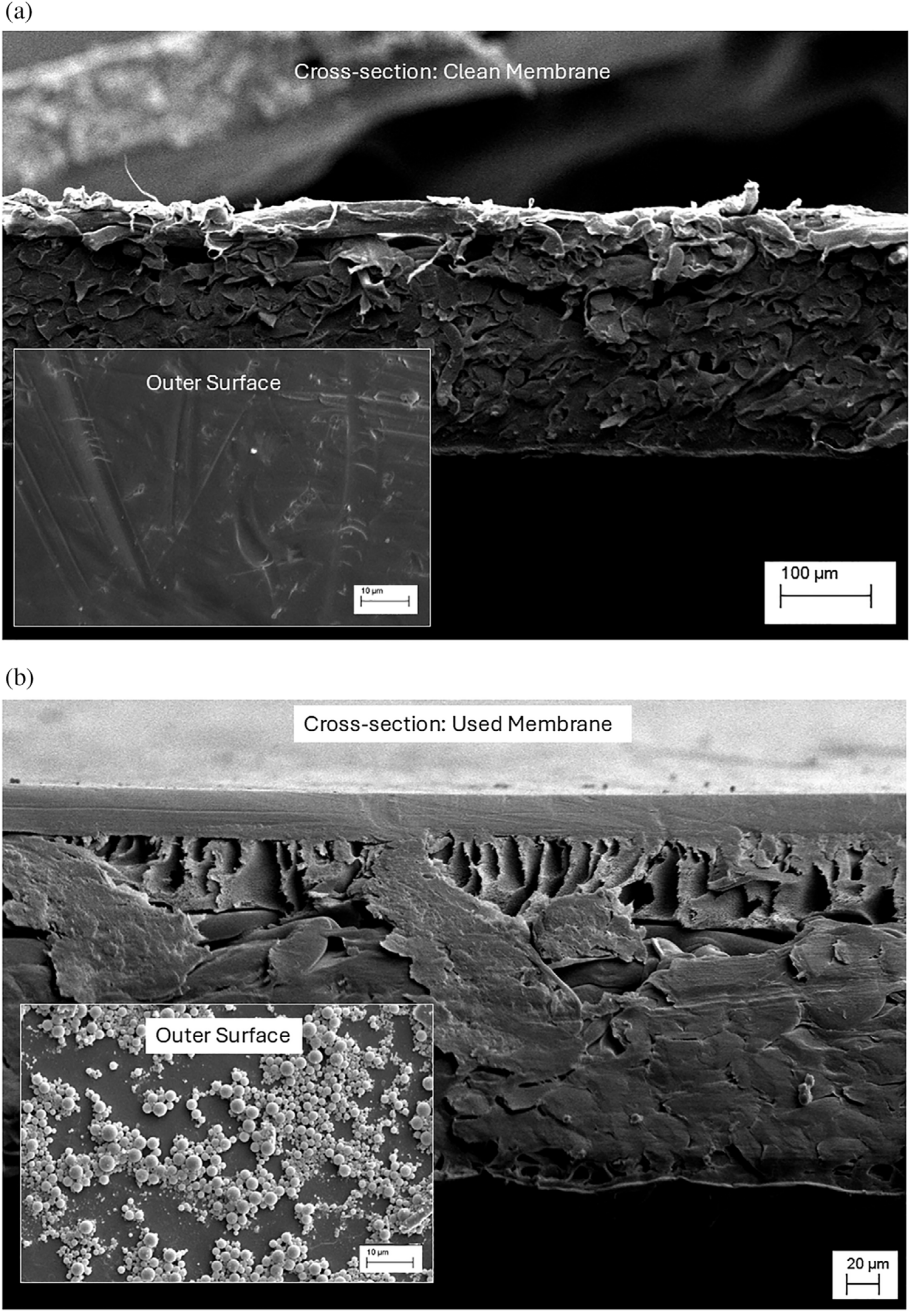
SEM images of the membrane (a) before and (b) after annatto seed extract filtration.

The formation of this dense cake layer contributed to the retention of bixin and other low‐molecular‐weight compounds by providing an additional selective barrier to solute transport. The deposited layer can act as an additional selective barrier, increasing the resistance to solute transport and reducing the effective passage of bixin through the membrane. This effect may occur together with the previously discussed aggregation of bixin and its association with other components of the annatto extract. Therefore, the high bixin rejection cannot be attributed exclusively to the intrinsic selectivity of the 5 kDa membrane but likely results from the combined effects of molecular aggregation, solute–membrane interactions, and the additional resistance imposed by the cake layer formed during filtration.

While cake formation contributed positively to bixin retention, its substantial thickness also increased hydraulic resistance and contributed to the observed permeate‐flux decline. In addition, the formation of a dense and compact cake layer may hinder membrane cleaning, reduce permeability recovery, and consequently compromise membrane reuse over successive filtration cycles. Therefore, alternative filtration strategies should be investigated to control excessive cake formation while preserving bixin retention. In particular, cross‐flow filtration, optimization of the transmembrane pressure and hydrodynamic conditions, and appropriate pretreatment of the feed could reduce particle accumulation at the membrane surface and improve process productivity.

The results presented in this study demonstrate the potential of integrating the extraction of bixin, phenolic compounds, and antioxidant compounds using an ethanol–water mixture with subsequent concentration of the extract by ultrafiltration. The use of ethanol instead of more hazardous organic solvents, such as acetone or chloroform, offers important advantages from the perspective of food safety and represents a potentially less hazardous solvent choice for the extraction process. Considering that the 5 kDa polyethersulfone ultrafiltration membrane does not provide selective separation between ethanol and water, both permeate and concentrate streams are expected to maintain an ethanol concentration close to that of the feed solution (50 vol%). However, the residual ethanol concentration was not experimentally quantified in the present study, and no specific solvent‐removal step was performed after ultrafiltration. Therefore, before the concentrated extract can be considered for food, pharmaceutical, or cosmetic applications, further analyses should be conducted to quantify the residual ethanol and to confirm that the extract complies with the safety and regulatory requirements applicable to each intended use. From an economic perspective, the integration of ethanol–water extraction with ultrafiltration may be attractive for scale‐up because membrane concentration operates at relatively low pressure and does not require a phase change, potentially reducing energy consumption compared with thermal concentration processes. Previous techno‐economic studies on annatto processing have demonstrated the potential economic feasibility of ethanol‐based extraction processes [5].

## 4 Conclusion

This study investigated the extraction of bioactive compounds from annatto (*B. orellana* L.) seeds, followed by their concentration through ultrafiltration. The chosen water–ethanol mixture for extraction proved effective in recovering both phenolic and carotenoid compounds. The resulting extract contained 1.91 ± 0.02 mg g^−1^ of bixin and 52.31 ± 2.45 mg GAE g^−1^ of TPC on a dry basis. The subsequent application of a dead‐end ultrafiltration process, utilizing a 5 kDa polyethersulfone membrane, promoted the concentration of bixin while allowing partial permeation of phenolic compounds. The membrane exhibited a high bixin rejection of 97.90 ± 0.02%, while the bixin concentration increased from 1.91 ± 0.02 to 2.47 ± 0.12 mg g^−1^ on a dry basis, corresponding to an EF of 1.29. In comparison, TPC showed a lower membrane rejection of 47.53 ± 0.22%. Analysis of the fouling mechanisms using the Hermia model revealed that cake formation was the predominant type of fouling during the initial times of the ultrafiltration process, while all fouling mechanisms contributed to the flux decline at the later stages of filtration. The permeate flux decreased to a steady‐state value of 9.26 ± 0.62 L h^−1^ m^−2^, and cake formation accounted for 55.4% of the total fouling resistance. The ultrafiltration process was efficient in concentrating the bixin molecules, while the TPC were partially retained by the membrane. These quantitative results demonstrate the selective character of the proposed process, particularly for the production of bixin‐rich annatto extracts. Further studies addressing solvent recovery, fouling control, process scale‐up, and the safety and regulatory requirements of the final extract are required before its application in food, pharmaceutical, or cosmetic products.

## Author Contributions

**Karolyna Barros Miguel**: conceptualization, investigation, writing – original draft, methodology, validation, formal analysis. **Vicelma Luiz Cardoso**: conceptualization, supervision, writing – review and editing. **Miria Hespanhol Miranda Reis**: conceptualization, supervision, writing – review and editing.

## Ethics Statement

The authors have nothing to report.

## Conflicts of Interest

The authors declare no conflicts of interest.

## Supporting information




**Supporting File**: cbdv71783‐sup‐0001‐SuppMat.docx.

## Data Availability

The data that support the findings of this study are available on request from the corresponding author.
