# Supplementary material for: Production of Bixin‐Rich Extracts From Annatto Seeds via Ethanol–Water Extraction Integrated With Ultrafiltration Technology
Source: Chem Biodivers. 2026 Sep 30;23(10):e71783. doi: 10.1002/cbdv.71783 (PMC13626603; doi:10.1002/cbdv.71783)
Supplement: Supplementary file 1 [file CBDV-23-e71783-s001.docx]

**Supplementary Information**

**Production of Bixin-Rich Extracts from Annatto Seeds via Ethanolic Extraction
Integrated with Ultrafiltration Technology**

Karolyna B. Miguel¹, Vicelma Luiz Cardoso¹, Miria Hespanhol Miranda Reis¹,*

¹ Chemical Engineering Faculty, Federal University of Uberlandia, Av. João Naves de Ávila 2121, Santa Mônica, Uberlândia, MG, 38408-144, Brazil

** Corresponding author: miria@ufu.br*

**HPLC chromatograms**

Figure S1 shows representative chromatograms of the bixin standard solutions used to construct the HPLC calibration curve. Under the chromatographic conditions described in the manuscript, bixin was detected at a retention time of approximately 13.9 min.

Figure S1. Representative HPLC chromatograms of the bixin standard solutions used for construction of the calibration curve. The dashed line indicates the bixin retention time (Rt ≈ 13.9 min).

Representative chromatograms of the feed, concentrate (retentate), and permeate samples are presented in Figure S2. Figure S3 provides an enlarged view of the chromatographic region associated with the bixin peak, facilitating comparison among the three streams.

Figure S2. Representative HPLC chromatograms of the annatto seed extract streams: feed, concentrate (retentate), and permeate after ultrafiltration with the 5 kDa polyethersulfone membrane.

Figure S3. Enlarged view of the chromatographic region associated with the bixin peak for the feed, concentrate (retentate), and permeate samples.
